# Spinal Subdural Hematoma Secondary to Ruptured Internal Carotid Artery Ophthalmic Segment Aneurysm: A Case Report and Narrative Review

**DOI:** 10.7759/cureus.66283

**Published:** 2024-08-06

**Authors:** Bilal Ibrahim, Maher Al-Khawaldeh, Lina A Abu Sirhan, Abdallah Arabyat, Rahmeh A Abdallah, Mohammad Y Hiasat, Mustafa Nadi, Waleed F Dabbas

**Affiliations:** 1 Faculty of Medicine, Division of Neurosurgery, Department of Special Surgery, Al-Balqa Applied University, Al-Salt, JOR; 2 Department of Interventional Neuroradiology, Jordanian Royal Medical Service, Amman, JOR; 3 Faculty of Medicine, Al-Balqa Applied University, Al-Salt, JOR; 4 Division of Neurosurgery, Department of Special Surgery, Neuron Clinics, Amman, JOR

**Keywords:** anterior cerebral aneurysms, internal carotid artery, cerebral aneurysms, spinal subdural hematoma, back pain

## Abstract

Spinal subdural hematoma (SSDH) is a rare condition where the exact pathology is unclear; coagulopathy, bleeding disorders, trauma, and iatrogenic causes are frequently associated with SSDH. SARS-CoV-2 infection and COVID-19 vaccines are unusual causes of SSDH, as reported by multiple studies. Here, we present a rare case report and a narrative review of SSDH resulting from a ruptured cerebral aneurysm.

A 53-year-old female presented with an acute, severe suboccipital headache and neck and back pain without radiculopathy. Investigations for cardiovascular diseases and brain images were unremarkable. Further investigation revealed an SSDH extending from T1 to S2. Negative spinal angiography led to a cerebral angiogram, identifying an internal carotid artery ophthalmic segment aneurysm that was successfully treated with endovascular stent-assisted coiling.

This case scenario of anterior circulation cerebral aneurysmal rupture manifesting as an isolated SSDH is unique compared to previously reported cases of SSDH resulting from cerebral aneurysms. This case highlights the importance of considering aneurysmal rupture in SSDH cases with no apparent underlying pathology to prevent neurological deficits. Early detection and intervention in such cases can prevent serious neurological deficits and improve patient outcomes.

## Introduction

Spinal subdural hematoma (SSDH) is a rare and complex condition, comprising approximately 5% of all intraspinal hematomas [1,2]. Etiology is multifactorial, with various causes such as trauma, iatrogenic factors, coagulopathy, and underlying autoimmune diseases. Coagulopathy, often resulting from underlying coagulation abnormalities or anticoagulant therapy, is thought to be the most common underlying cause of SSDH [3].

It has been reported in the literature that SARS-CoV-2 infection can cause hemorrhagic spinal lesions like spontaneous SSDH. Furthermore, COVID-19 vaccines were associated with nervous system complications such as cerebral venous thrombosis, intracerebral hemorrhage, and hemorrhagic spinal lesions [4,5].

In this case report, we present a unique instance of an isolated SSDH caused by an intradural internal carotid artery (ICA) aneurysm rupture. It is distinct from previously documented cases that were associated with subarachnoid hemorrhage (SAH). The patient presented with a sudden, severe occipital headache followed by severe back pain, accompanied by vision disturbance and vomiting. Multiple diagnostic workups, brain CT, and MRI revealed no abnormalities.

## Case presentation

A 53-year-old female, known to have hypertension and to be a smoker, presented with a sudden severe occipital headache while driving. The pain radiated to the neck and shoulder blades and was accompanied by vision disturbance and vomiting. The patient was not on any anticoagulant or antiplatelet medications. Upon arrival, her Glasgow Coma Scale was 15/15. Neurological examination revealed intact reflexes and sensation, normal gait and posture, and no cranial nerve deficits. However, meningeal signs (Kernig's and Brudzinski's) were positive. We admitted the patient to the neurosurgery ward.

She underwent multiple diagnostic workups, including brain CT and MRI, which detected no abnormalities. In the next few days, the patient started to experience progressive, severe middle and lower back pain without radiculopathy. Consequently, we conducted a whole spine MRI for further investigation.

**Figure 1:**
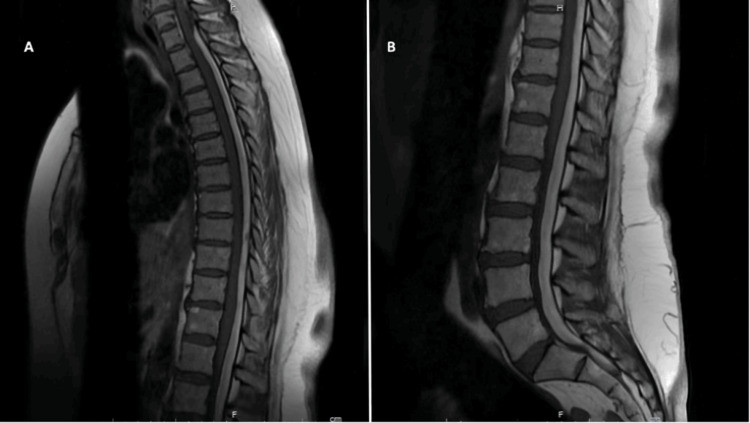
MRI T1 weighted sagittal view of the thoracic (A) and lumbar (B) spine showing acute SSDH MRI: magnetic resonance imaging, SSDH: spinal subdural hematoma

**Figure 2:**
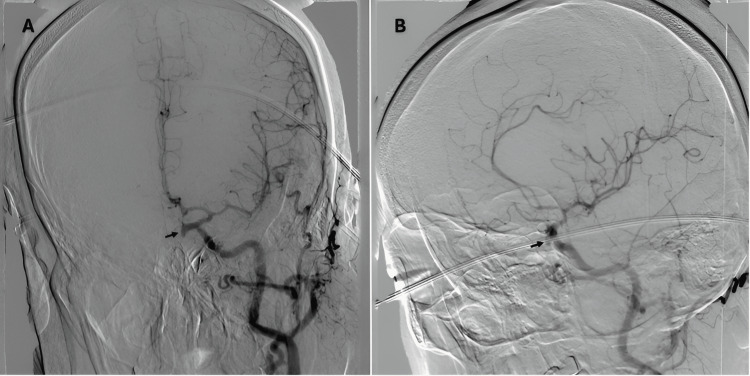
Anteroposterior (A) and lateral (B) views of an ICA angiography showing an inferomedially projecting ophthalmic segment saccular aneurysm. This aneurysm has a height of 6 mm, a maximal diameter of 3.2 mm, a neck width of 2 mm, and a dome-to-neck ratio of 1.6 mm

Thoracolumbar MRI revealed subdural hematoma at the posterior aspect of the spinal cord, extending from T1 to S2 levels, with a maximum width of 8 mm in the lumbar region (Figure 1). Suspecting a spinal vascular malformation as the underlying cause, a conventional spinal angiography was performed, which showed no vascular malformations. Hence, a cerebral angiogram was done to investigate the vascular causes of the SSDH. It showed an elongated saccular aneurysm of the left ophthalmic segment of the ICA aneurysm, projecting inferomedially. The aneurysm height is 6 mm, its maximal diameter is 3.2 mm, its neck width is 2 mm, and the dome-to-neck ratio is 1.6 mm (Figure 2).

**Figure 3:**
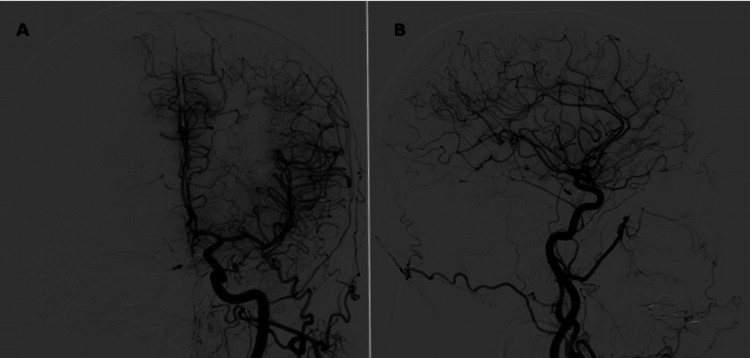
One year post-coiling of left ICA angiography showing an aneurysm occlusion with no recanalization (arrow) ICA: internal carotid artery

After discussing management options with the patient, therapeutic endovascular stent-assisted coiling of the aneurysm was performed as the patient refused craniotomy and surgical clipping, and anticoagulants were subsequently initiated. Follow-up cerebral angiography conducted at three months and one year post-coiling showed aneurysm occlusion with no recanalization (Figure 3).

## Discussion

SSDH represents a rare and intricate condition, making up approximately 5% of intraspinal hematomas [1,2]. The causes of SSDH are varied, involving trauma, bleeding disorders, autoimmune diseases, and iatrogenic causes. Coagulopathy, frequently associated with coagulation defects or anticoagulant treatment, is the most prevalent underlying cause of SSDH [3]. COVID-19 and COVID-19 vaccines are the most recent and updated causes of SSDH [4,5].

The patient presented with an acute, severe occipital headache and tearing neck and back pain without radiation. Initial brain imaging was unremarkable, and cardiovascular pathologies were considered but ruled out after an extensive workup. Further investigation revealed an SSDH extending from T1 to S2. Negative spinal angiography led to a cerebral angiography, identifying an ICA ophthalmic segment aneurysm that was successfully treated with endovascular stent-assisted coiling.

Ophthalmic artery aneurysms (OAAs) occur in 5% to 9% of anterior circulation aneurysms. They have been reported to be more common in females and are associated with other aneurysms in around 20% of cases [6]. OAAs have slower growth and a lower risk of rupture [7,8].

Unlike most other aneurysms, OAAs are more controversial to manage, especially regarding technique selection and long-term outcomes. Microsurgery approaches provide great anatomical visualization, safe vascular exposure, and effective postoperative outcomes [9].

OAAs have complex, tortuous vascular anatomy, making endovascular treatment challenging [10]. Endovascular treatment of these aneurysms is associated with higher rates of incomplete aneurysm occlusion and recurrence and may not address the optic nerve compression symptoms that microsurgery can. Additionally, endovascular treatment requires prolonged use of potent antiplatelet drugs, limiting their use in acute aneurysm rupture and posing bleeding complications [11]. Endovascular approaches also carry a risk of severe morbidity and mortality if the vessel ruptures [12].

OAA microsurgery is a safe and effective procedure that improves the visual and neurological status. In addition, OAA can be clipped successfully with excellent results; however, postoperative visual deficits are a concern, and errors in their execution can lead to visual deficits, catastrophic bleeding, or strokes [9]. Similarly, the visual outcomes following endovascular treatment are also a concern [13].

The patient was presented with the two primary treatment options for her cerebral aneurysm. After a thorough discussion of the risks and benefits of each approach, including the recanalization rate after endovascular coiling, the patient opted for endovascular coiling due to its less invasive nature compared to craniotomy and surgical clipping.

**Figure 4:**
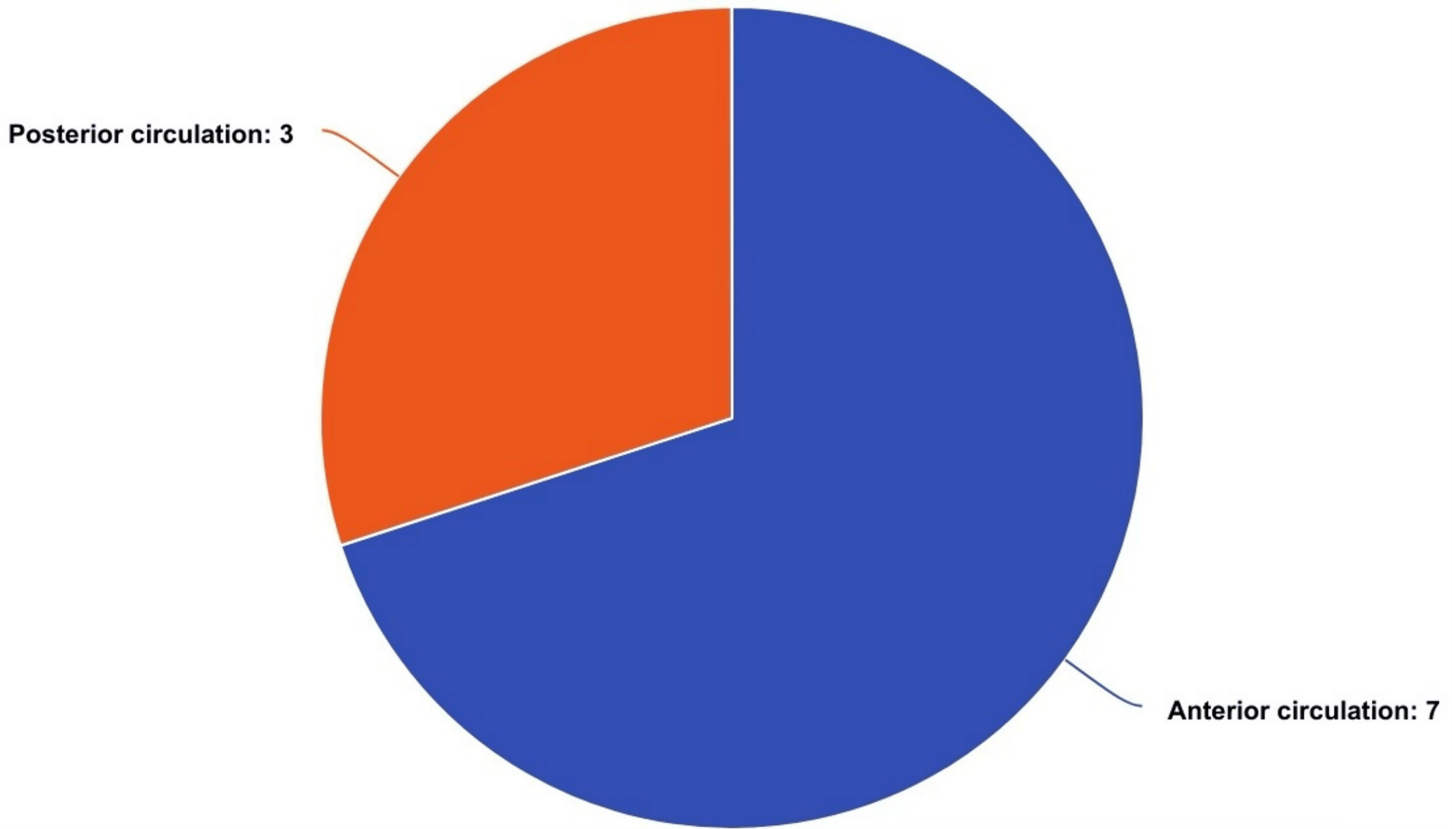
Distribution of aneurysms in the reported cases including our case

This presentation of anterior circulation cerebral aneurysmal rupture manifesting as an isolated SSDH is unique compared to previously reported cases of SSDH resulting from cerebral aneurysms. It is the first documented case in the literature where the patient presented solely with a spinal subdural hemorrhage due to a ruptured cerebral aneurysm without any intracranial hemorrhage. A review of nine documented cases of SSDH due to ruptured intracranial aneurysms revealed that the aneurysms were located in various cerebral arteries (Table 1, Figure 4).

**Table 1:** Reported cases of SSDH caused by rupture of intracranial aneurysms MCA: middle cerebral artery, ACA: anterior cerebral artery, ICA: internal carotid artery, PCA: posterior cerebral artery, PICA: posterior inferior cerebellar artery, SSDH: spinal subdural hematoma

	Study	Age (years), sex	Location of aneurysm	Presence of brain SAH	SSDH discovery after SAH	Level of SSDH	The patient on anticoagulant\antiplatelets	SSDH treatment	Aneurysm treatment
1	Kim et al., 2007 [2]	50, M	ACA	Yes	SAH day 4	T11-L5	Not specified	Conservative	Clipping
2	Kim et al., 2007 [2]	39, F	MCA bifurcation	Yes	SAH day 9	L4-L5	Not specified	Conservative	Clipping
3	Kim et al., 2007 [2]	60, F	ACA	Yes	SAH day 10	S1	Not specified	Conservative	Clipping
4	Waldron et al., 2011 [14]	37, F	Left intracranial Vertebral artery	Yes	SAH day 6	T12- S1	Not specified	laminectomies of L4-S1	Coil embolization
5	Yamaguchi et al., 2003 [15]	52, F	Left ICA	Yes	SAH day 6	Lumbosacral	No	Conservative	Clipping
6	Rothrock 2019 [16]	66, F	Basilar tip, left MCA, left PICA, right ICA, right MCA	Yes	SAH day 3	L1-L5	No	Bilateral laminectomies of L1-L4	Coil embolization
7	Choi et al., 2016 [17]	52, M	ACA	Yes	SAH day 3	L4-S2	Not specified	Left hemilaminectomies of L4-S1	Coil embolization
8	Ovalı et al., 2014 [18]	55, F	V4 vertebral artery	Yes	SAH day 21	T10-S2	Not specified	Conservative	Coil embolization
9	Kobayashi et al., 2007 [19]	62, M	Right PCA	Yes	SAH day 2	L4-S2	No	Conservative	Clipping
10	Current study	53, F	ICA ophthalmic segment	No	-	T1-S2	No	Conservative	Stent-assisted coiling

Patients with SSDH typically present with back pain and radiculopathy, often accompanied by motor and sensory deficits, with the thoracic/thoracolumbar spine being the most common location of the back pain [20]. The typical manifestation of SSDH is back pain, commonly associated with motor and/or sensory deficits. De Beer et al. found in a systematic review that 45% of SSDH cases were associated with coagulopathy. In our presenting case, the patient was not on anticoagulants or antiplatelet medications.

Untreated, symptomatic SSDH is associated with high morbidity, emphasizing the importance of early recognition and management. Surgical decompression of the spinal cord in patients with complete spinal cord lesions has a dismal outcome, whereas, for incomplete spinal cord lesions, the outcomes are more promising [21]. In the review by De Beer et al., 51% of surgically treated patients were ambulatory. In our case, the patient complained of severe back pain without neurological deficits, which was controlled with analgesics and did not require surgical intervention. The patient remained neurologically intact throughout treatment and at follow-up after 10 weeks of discharge.

The exact pathophysiology of blood extension from the intracranial subarachnoid space to the spinal subdural space remains unclear. However, theories propose that aneurysmal adherence to arachnoid granulations can lead to small tears, allowing recurrent microbleeding and blood entry into the subdural space. Additionally, increased pressure can cause tears in the pia-arachnoid, permitting blood to leak into the subdural space [22].

## Conclusions

An ophthalmic segment of the internal carotid artery aneurysm causing an isolated SSDH is rare. Early diagnosis of atypical presentations of SSDHs is crucial for determining a patient's functional outcome. In cases where an underlying pathology is not readily apparent, it is essential to consider the possibility of aneurysmal rupture to prevent the development of neurological deficits and to ensure an accurate diagnosis and effective management.
